# Optical coherence tomography detection of changes in inner retinal and choroidal thicknesses in patients with early retinitis pigmentosa

**DOI:** 10.5935/0004-2749.20200080

**Published:** 2024-02-11

**Authors:** Fahrettin Akay, Berkay Akmaz, Yusuf Ziya Güven

**Affiliations:** 1 Department of Ophthalmology, İzmir Katip Çelebi University Atatürk Training and Research Hospital, İzmir, Turkey

**Keywords:** Choroid/anatomy & histology, Retina/anatomy & histology, Retinal ganglion cell, Retinitis pigmentosa, Tomography, optical coherence, Coroide/anatomia & histologia, Retina/anatomia & histologia, Células ganglionares da retina, Retinite pigmentosa, Tomografia de coerência óptica

## Abstract

**Purpose:**

To evaluate the inner retinal and choroidal thicknesses in patients with early retinitis pigmentosa.

**Methods:**

We analyzed spectral-domain optical coherence tomography images of 35 retinitis pigmentosa patients and 40 healthy individuals. We measured macular and ganglion cell complex thicknesses. We took choroidal thickness measurements in the subfoveal region and 500, 1,000, and 1,500 µm from the foveal center.

**Results:**

Patients with retinitis pigmentosa had significantly thinner macular thicknesses and choroidal thicknesses in all measurements, and their individual ganglion cell complex thickness measurements were lower than those in healthy individuals. The mean ganglion cell complex thickness was significantly lower in patients with retinitis pigmentosa than that in controls. The mean macular thickness was significantly correlated with the mean choroidal and mean ganglion cell complex thicknesses. (We found no correlation between the mean choroidal thickness and the mean ganglion cell complex thickness).

**Conclusions:**

The choroid was mildly affected in our patients with early retinitis pigmentosa. The tendency toward significance in the inner retina was possibly caused by a good visual acuity.

## Abstract (pt)

**Objetivo:**

Avaliar as espessuras internas da retina e da coroide em pacientes com retinite pigmentosa precoce.

**Métodos:**

Foram analisadas imagens de tomografia de coerência óptica de domínio espectral de 35 pacientes com retinite pigmentosa e 40 indivíduos saudáveis. Medimos a espessura do complexo de células maculares e ganglionares. Realizamos medições da espessura da coroide na região subfoveal e a 500 µm, 1000 µm e 1500 µm do centro da fóvea.

**Resultados:**

Pacientes com retinite pigmentosa apresentaram espessuras maculares e da coroide significativamente mais finas em todas as medições e suas medidas individuais da espessura do complexo de células ganglionares foram inferiores às de indivíduos saudáveis. A espessura média do complexo de células ganglionares foi significativamente menor nos pacientes com retinite pigmentosa do que nos controles. A espessura macular média foi significativamente correlacionada com as espessuras médias do complexo das células de coroide e das células ganglionares médias. Não encontramos correlação entre a espessura media da coroide e a espessura media do complexo de células ganglionares.

**Conclusões:**

A coroide foi levemente afetada em nossos pacientes com retinite pigmentosa precoce. A tendência à significância na retina interna foi possivelmente causada por uma boa acuidade visual.

## INTRODUCTION

The term retinitis pigmentosa (RP) encompasses a group of inherited retinal diseases with progressive retinal degeneration, characteristically starting in the mid-periphery and advancing toward the macula and fovea^(1)^. RP is associated with nyctalopia and progressive peripheral visual field losses, followed by reductions in central vision and electroretinogram (ERG) abnormalities due to degeneration and loss of photoreceptors and the retinal pigment epithelium (RPE)^(1,2)^.

Spectral-domain optical coherence tomography (SD-OCT) is the standard noninvasive and sensitive imaging method to monitor the natural progression of RP^(3-5)^. It can also accurately reveal the internal architecture and the structural changes in several retinal layers^(3,4)^. SD-OCT images have a predictable association with histology in animal models, and studies have revealed the potential of SD-OCT for monitoring structural progression of degenerating retinas^(6)^.

Studies have reported retinal and choroidal thicknesses (ChTs) in patients with RP and have concluded that the outer retinal microlayers are primarily responsible for losses in visual acuity (VA)^(7-9)^. Histopathological studies have revealed reduced rod and cone cells and thinning of the outer photoreceptor layer^(1,2)^. Other studies have also suggested that retinal thickness as well as the status of the ellipsoid zone (inner segment/outer segment junction) of photoreceptors, the external limiting membrane (ELM), and cone outer segment tips (COSTs) are significantly correlated with VA in patients with RP^(3-5,9-12)^. Reductions in the ellipsoid zone and the fundus autofluorescence ring are significantly correlated with VA and decreases in retinal sensitivity^(4,5,10)^. The inner retina has been found to be impaired because of transneural damage, vascular compromise, or axonal compression secondary to thinning of the outer retina^(13)^. Almost all other studies about changes in retinal thickness and ChT have included patients with RP with an impaired VA. Optic nerve head pallor has led to the clinical conclusion that RP causes transneuronal degeneration of ganglion cells following death of the photoreceptors^(13)^. Optic nerve head pallor is an objective finding signaling the injury of inner retinal layers. However, no studies have investigated inner retinal layer thickness changes using SD-OCT or the association between the inner retinal thickness and ChT with central retinal functioning in patients with RP.

Therefore, we characterized changes in the thicknesses of the macula, choroid, and ganglion cell complex (GCC) in patients with RP with normal VA and assessed associations between changes in GCC and ChT and disease severity.

## METHODS

We designed a prospective case-control study, and the ethics committee of our institution approved it. We reviewed medical records of 35 patients with RP with normal VA. We included the records of 40 age-matched healthy subjects for a control group. All patients with RP were enlisted men in the Turkish army. Healthy applicants for employment in the army (all men) constituted the control group. The study adhered to the tenets of the Declaration of Helsinki.

We based RP diagnoses on findings such as night blindness history, restricted peripheral vision as assessed using the visual field test, reduced aand b-waves using a full-field ERG, and pathognomonic fundus appearance (attenuated vessels and bone-spicule pigment clumping). We excluded participants with spherical refractive errors higher than ±3.00 diopters and/or astigmatism higher than ±3.00 in any eye; those with significant opacities precluding fundus examination or imaging of the refractive media; those with ocular hypertension or glaucoma; those with a history of optic neuropathy, disk drusen, uveitis, or any type of previous retinal disease or treatment; those with previous refractive/intraocular surgery; those with atypical RP (sector RP and unilateral RP); those with neurodegenerative disorders; those smoking; those using medications affecting visual and/or retinal function/thickness; and those with other local or systemic diseases. In addition, we excluded patients with other subtypes of RP like syndromic RP (Usher syndrome) and congenital stationary night blindness.

Each participant underwent a complete ophthalmic examination, including best-corrected Snellen VA (BCVA) converted to the logarithm of minimal angle of resolution, slit-lamp stereo biomicroscopy, and intrao cular pressure (IOP) measurements using Goldman applanation tonometry (Haag-Streit, Bern, Switzerland). We measured axial lengths (ALs) using a biometer (OcuScan; Alcon, Fort Worth, TX, USA). All participants underwent a 24-2 visual field test (Octopus 900, Haag-Streit) and full-field ERG (Metrovision, Pérenchies, France). We used mean defect (MD) and square root of loss variance (sLV) values for statistical evaluations. We performed dilated fundus examinations via indirect ophthalmoscopy.

**Figure 1:**
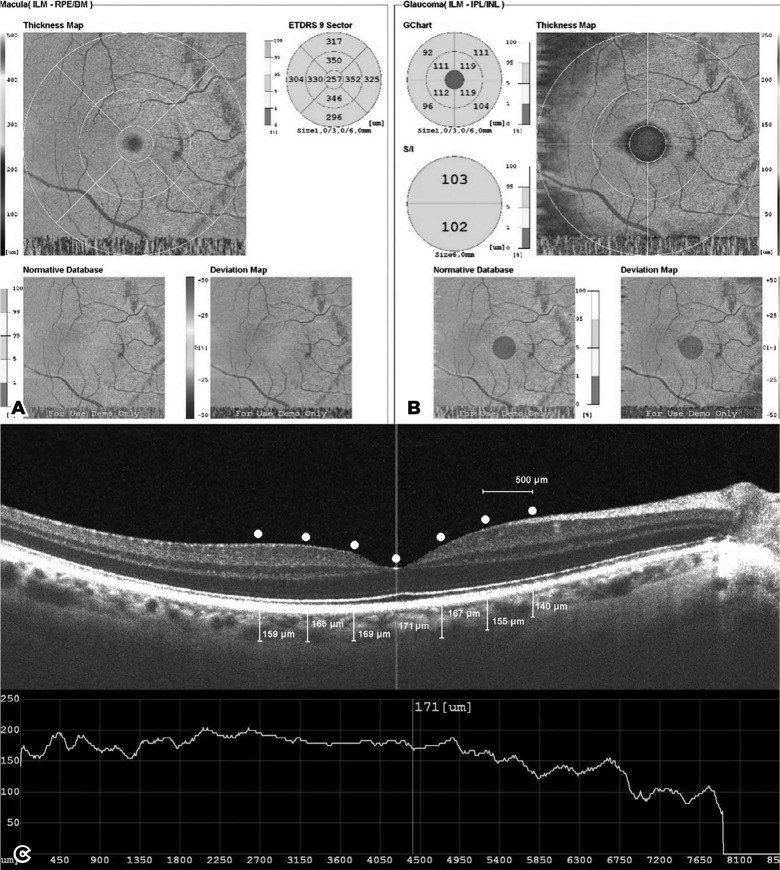
Representative spectral-domain optical coherence tomography images. We took macular thickness and ganglion cell complex thickness measurements using the Macula Map protocol. A) shows the nine regions where the macular thickness was measured. The measurements were performed in the central foveolar region and in the inner 3 mm and outer 6 mm rings around the foveolar region. We divided the inner and outer rings into four quadrants defining the temporal, nasal, superior, and inferior areas. B) shows the eight areas of ganglion cell complex thickness measurements. We took measurements in the four quadrants in both the inner and outer rings. C) shows choroidal thickness measurements under the enhanced depth-imaging mode in the subfoveal and 500, 1,000, and 1,500 _µ_m nasal and temporal from the foveal center.

We obtained retinal and choroidal SD-OCT images using the RS-3000 apparatus (Nidek, Gamagori, Japan) after pupil dilation with 2.5% phenylephrine hydrochloride and 1% tropicamide and used the Macula Map image protocols. We obtained three images from each participant, and chose the one with the highest signal strength for analyzes. The Macula Map scan pattern evaluated a 6 × 6 mm area centered on the fovea with 64 horizontal B-scan lines, each consisting of 1,024 A-scans per line. The retinal thickness was automatically calculated in nine Early Treatment Diabetic Retinopathy Study (ETDRS) areas, consisting of a central circular zone with a 1 mm diameter, representing the foveal area (central macular thickness [CMT]) and 3 mm inner and 6 mm outer diameter rings. The inner and outer rings were divided into four quadrants. The mean retinal thickness and the mean thickness of each of the five retinal layers in each of the nine ETDRS subfields were recorded (Figure 1A). We used the glaucoma tab (same setting) to view the GCC thickness map (ILM-IPL/INL). We measured the average GCC thickness in four quadrants of each inner and outer ring (circular zones with 3 mm inner and 6 mm outer rings, divided into four quadrants) (Figure 1B). We measured ChT as the perpendicular distance between the outer border of the RPE and the scleral interface (Figure 1C). We also obtained ChT measurements of the subfoveal nasal (500, 1,000, and 1,500µm to the fovea) and temporal (500, 1,000, and 1,500µm to the fovea) regions.

Before beginning the study, we used the values of ChT obtained with a Nidek RS-3000 SD-OCT in healthy subjects (mean ± standard deviation, 329.5 ± 65.2 µm) reported by Vujosevic et al.^(14)^ to calculate the required sample size for the study based on an alpha error of 5% and a beta error of 80%. The calculated minimum required sample size was 26 patients. We analyzed all statistical data using the SPSS statistical software for Windows, version 21.0 (SPSS, Chicago, IL, USA). Values are expressed as the means ± standard deviations. We only selected data from the right eye of each participant for statistical analyzes to avoid compromising the independence of the variables; we considered all *P-values <*0.05 as statistically significant. We analyzed normality of the values using the Shapiro-Wilk test and applied either the independent samples *t* test or the Mann-Whitney U test according to the Shapiro-Wilk test results. We considered differences as significant at p-values <0.05. We investigated correlations among variables using Pearson’s or Spearman’s correlation coefficients.

## RESULTS

We found no significant differences in terms of age, BCVA, refractive error, IOP, or AL measurements between the groups (p>0.05). Table 1 shows the demographic, clinical, and visual field data for all participants.

**Table 1:** Demographic, ocular, and visual field parameters of patients with retinitis pigmentosa and of the control group

Parameters	Retinitis pigmentosa (n = 35)	Controls (n=40)	p-value
Age (years)	22.9 ± 1.9	22.8 ± 1.8	0.817^*^
1OP (mmHg)	15.7 ± 2.9	15.4 ± 2.3	0.185+
Axial length (mm)	23.2 ± 0.9	23.1 ± 0.8	0.509+
Spherical equivalent (dpt)	-0.36 ± 0.78	-0.2 ± 0.82	0.448†
BCVA (logMAR)	0.03 ± 0,04	0.02 ± 0.04	0,399+
MD (dB)	14.6 ± 1,2	N/A	-
sLV (dB)	10.8 ± 1,6	N/A	-

IOP= intraocular pressure; BCVA= best corrected visual aquity; logMAR= logarithm of the Minimum Angle of Resolution; MD= mean defect; sLV= square root of loss variance; dB= decibel; N/A= not applicable.

Values are presented as means ± SDs.

* Mann-Whitney U test,

† Independent samples t test.

Patients with RP had significantly thinner MTs in all measurement areas (Table 2). The subfoveal ChT was the thickest region, and the ChT decreased gradually toward the peripheral retina in both the patients with RP and the control individuals. Similarly, patients with RP had significantly thinner choroids than control individuals in all measurement areas. Both patients with RP and control individuals had lower ChTs in the nasal to the foveal region than those in the temporal to the foveal region (Table 3).

**Table 2:** Macular thickness and ganglion cell complex thickness differences between patients with retinitis pigmentosa and the control group

Location	Retinitis pigmentosa (n=35)	Controls (n=40)	p-value^*^
Foveal MT	245.7 ± 21.5	260.5 ± 21.6	<0.001
Mean MT	299.6 ± 14.7	324.3 ± 15.9	<0.001
Inner temporal MT	326.1 ± 17.0	336.0 ± 16.9	0.018
Inner nasal MT	330.0 ± 17.0	350.8 ± 28.2	0.001
Inner superior MT	337.9 ± 15.3	355.1 ± 26.0	0.001
Inner inferior MT	326.5 ± 23.8	361.5 ± 23.9	<0.001
Outer temporal MT	255.4 ± 35.6	311.3 ± 40.1	<0.001
Outer nasal MT	317.6 ± 12.6	328.1 ± 28.9	0.044
Outer superior MT	295.9 ± 20.3	319.2 ± 32.2	0.001
Outer inferior MT	261.3 ± 50.1	296.1 ± 17.3	0.043
Mean GCC	107.7 ± 8.3	112.0 ± 7.2	0.019^†^
Inner temporal superior GCC	109.7 ± 7.9	113.3 ± 8.2	0.054^†^
Inner temporal inferior GCC	108.8 ± 19.3	114.3 ± 7.9	0.146^*^
Inner nasal superior GCC	118.8 ± 9.9	122.8 ± 9.1	0.069^†^
Inner nasal inferior GCC	118.9 ± 9.7	122.5 ± 8.1	0.085^†^
Outer temporal superior GCC	87.5 ± 11.8	91.3 ± 10.8	0.150^†^
Outer temporal inferior GCC	89.7 ± 12.5	95.1 ± 11.9	0.059^†^
Outer nasal superior GCC	112.2 ± 20.8	117.2 ± 10.1	0.303^*^
Outer nasal inferior GCC	116.2 ± 12.5	119.5 ± 11.4	0.184^*^

MT= macular thickness; GCC= ganglion cell complex;

* Mann-Whitney U test=

† Independent samples t test. Values are presented as means ± SDs.

**Table 3:** Choroidal thickness differences between patients with retinitis pigmentosa and control individuals

Location	Retinitis pigmentosa (n=35)	Controls (n=40)	p-value^*^
Subfoveal	233.7 ± 26.1	278.2 ± 34.4	<0.001
Mean	219.5 ± 23.6	272.3 ± 33.0	<0.001
Nasal-500	225.6 ± 26.0	275.5 ± 35.8	<0.001
Nasal-1000	205.8 ± 23.2	267.6 ± 34.7	<0.001
Nasal-1500	194.6 ± 25.0	254.1 ± 32.9	<0.001
Temporal-500	229.9 ± 23.8	279.6 ± 35.1	<0.001
Temporal-1000	224.5 ± 25.4	278.5 ± 33.1	<0.001
Temporal-1500	222.4 ± 26.7	272.4 ± 32.3	<0.001

* Independent samples t test.

Values are expressed as means ± standard deviations.

Patients with RP had thinner GCCs for all of the measurement points, but some of the differences between groups were nonsignificant; the mean difference was significant (Table 2).

**Figure 2:**
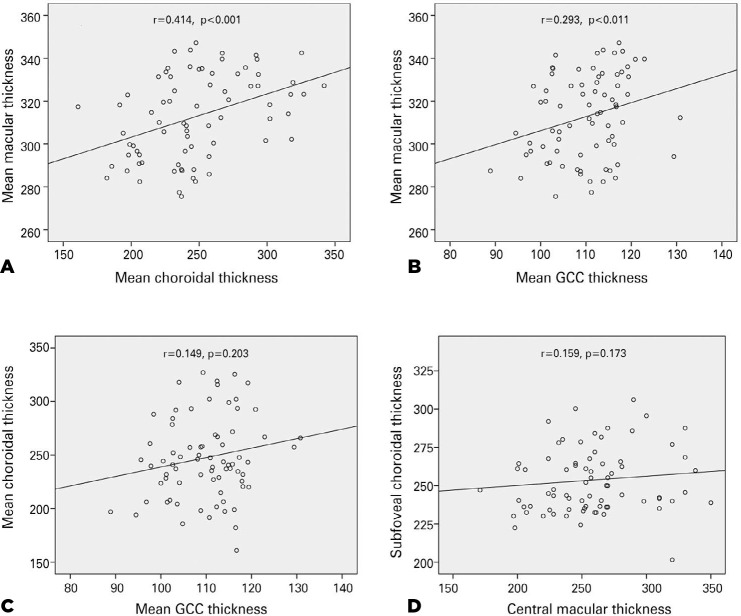
Correlations between the mean macular thickness and the mean choroidal thickness (A), between the mean macular thickness and the mean ganglion cell complex thickness (B), between the mean choroidal thickness and the mean ganglion cell complex thickness (C), and between the subfoveal choroidal thickness and the central macular thickness (D).

The mean MT was significantly correlated with the mean ChT and GCC thickness. However, we found no significant correlations between the mean ChT and the mean GCC thickness (Figure 2), nor between the mean subfoveal ChT and the mean CMT. We performed a Pearson’s correlation test with visual field test parameters (MD and sLV) and OCT measurements and found a negative correlation between the mean macular thickness and the visual field indices (MD, r=-0,872, p<0.001; sLV, r=-0,550, p=0.001, respectively).

## DISCUSSION

The ChT and macular thickness were reduced in patients with RP and normal VA. The mean GCC thickness was also significantly lower in patients with RP, although differences in measurements in individual areas were nonsignificant (almost significant in some locations). In addition, the MT was significantly and positively correlated with the ChT, but the correlation between the MT and the GCC thickness was nonsignificant.

Hemodynamic studies have demonstrated ocular blood flow disturbances in retrobulbar vessels as well as in the retina and choroid in patients with RP^(15-17)^. Such studies have also shown an association between increased plasma levels of endothelin-1 and decreased retinal and choroidal blood flows, even during the early stages of RP (before the appearance of abnormal ophthalmic symptoms)^(18-20)^. Endothelin-1 may reduce the ocular blood flow, which may lead to ischemic damage to both the optic nerve head and the retinal ganglion cells^(19)^. A mild choroidal thinning in inherited retinal diseases may be secondary to choriocapillaris thinning^(20)^. However, enhanced depth-imaging OCT cannot discriminate the choriocapillaris. Therefore, we could not determine whether the decrease ChT was secondary to choriocapillaris thinning.

To the best of our knowledge, Lucas^(21)^ was the first to report great ChT variance in the eyes of two patients with RP during an *in vitro* study. High-frequency ultrasound and partial coherence interferometry have been used to measure ChT in animals and humans^(22,23)^. Ho wever, a satisfactory choroidal interface cannot be detected in some patients using these techniques. SD-OCT provides sufficient resolution to see the choroidal-scleral interface *in vivo*.

Ayton et al reported significantly thinner choroids in patients with RP with mild to severe visual impairments than those in controls and reported significant negative correlations between the ChT and disease duration and VA^(24)^. They speculated that age might have been a confounding factor besides disease progression in the significant correlation between ChT and disease duration, as ChT was shown to decrease with age^(24)^. However, we believe that the significant correlation between ChT and VA in that study may indicate a direct association between ChT and disease progression and that the significant correlation between ChT and disease duration may not be only related to aging changes. In our study, we did not perform correlation analyzes between ChT and disease duration, because most patients were not sure of the exact date of their symptoms. In addition, the ages of participants were restricted to a small range. Similar to our findings, Dhoot et al found significantly lower subfoveal, nasal, and temporal ChTs in patients with RP^(25)^. In addition, we also found a lower nasal than a temporal ChT, a finding in line with that of Dhoot et al.^(25)^. Surprisingly, those authors reported a nonsignificant difference in the CMT between patients with RP and their controls. In contrast to Ayton et al.^(24)^, Dhoot et al reported a nonsignificant correlation between VA and subfoveal ChT^(25)^. In our study, the differences in ChT between patients with RP and control subjects were apparent, and all measurement points had values of p<0.001. The thinner choroids in patients with RP may be related to the decreased ocular blood flow reported^(25-27)^. Also, the thinner choroid may be related to the RPE and photoreceptor degeneration in patients with RP, which may result in choroidal thinning due to atrophy of the choriocapillaris^(26,27)^. In animal models, the RPE and photoreceptors have been shown to be needed for choroidal development and maintenance because they produce several factors such as vascular endothelial growth factor^(28)^.

Irrespective of these potential mechanisms, we expected a significant correlation between the retinal thickness and the ChT. In our study, the correlation between the mean MT and ChT was significant. We also found a weak positive correlation between the CMT and the subfoveal ChT, although not statistically significant. Dhoot et al.^(25)^ and Yeoh et al.^(20)^ did not find significant correlations between these parameters and inherited retinal diseases in patients with RP. However, as in our study, the correlation analyzes of Dhoot et al included all participants (personal communication); in addition, the findings of Yeoh et al could not be generalized to patients with RP, because only 2 in >20 patients had rod-cone dystrophy that could be RP subtypes. We believe that the significant correlations between the mean ChT and mean MT values are more valuable than the correlations between single measurements involving the CMT and the subfoveal ChT. However, larger longitudinal studies are necessary to confirm the exact associations between the subfoveal ChT and CMT in patients with RP.

Few other studies have investigated inner retinal changes in patients with RP: Battu et al showed that VA and retinal sensitivity are significantly correlated with the outer retinal structure and intact COST line, ELM, and ellipsoid zone but not with the overall thickness of the inner retina or ChT^(7)^. Aleman et al. reported outer nuclear layer thinning and inner retinal thickening in X-linked patients with RP with RP GTPase regulator mutations^(29)^. They speculated that the inner retinal abnormalities represented a detectable noninvasive marker for the neuronal-glial remodeling response secondary to photoreceptor stress or loss and suggested that intracellular or extracellular edema may have played a role in the increased thickness of the pericentral retina^(29)^. Similar findings have also been reported in Leber congenital amaurosis^(30)^. However, to the best of our knowledge, ours is the first study to investigate changes in GCC thickness in patients with RP, who had thinner GCCs than control subjects. Although not all subfield comparisons were significantly different, the mean GCC thickness was significantly lower in patients with RP. Moreover, the correlation between the mean MT and mean GCC thickness was significant, even though the correlation between the mean GCC thickness and mean ChT was nonsignificant. Also, we found a negative significant correlation between mean MT and visual field indices (MD and sLV). The visual field narrows as retinal damage progresses. These findings confirm visual function and structural changes in RP.

In the present study, we included patients with RP and normal VA to observe initial changes in the retina and choroid. Our results indicated 4% decreases in the mean GCC thickness, 6% decreases in the foveal MT, 7% decreases in the average MT, 16% decreases in the subfoveal ChT, and 19% decreases in the average ChT in patients during the early stages of RP and preserved central vision than in normal controls. Our results suggest that changes in ChT are more apparent than those in retinal thickness in patients with RP. In conclusion, RP resulted primarily in the progressive loss of rod and cone photoreceptors. ChT and GCC thickness were affected in patients with RP with normal central VA during the early stages of the disorder.
